# Triphasic production of IFN*γ* by innate and adaptive lymphocytes following influenza A virus infection

**DOI:** 10.1093/discim/kyad014

**Published:** 2023-08-19

**Authors:** George E Finney, Kerrie E Hargrave, Marieke Pingen, Thomas Purnell, David Todd, Freya MacDonald, Julie C Worrell, Megan K L MacLeod

**Affiliations:** Centre for Immunobiology, School of Infection and Immunity, University of Glasgow, Glasgow, UK; Centre for Immunobiology, School of Infection and Immunity, University of Glasgow, Glasgow, UK; Centre for Immunobiology, School of Infection and Immunity, University of Glasgow, Glasgow, UK; Centre for Immunobiology, School of Infection and Immunity, University of Glasgow, Glasgow, UK; Centre for Immunobiology, School of Infection and Immunity, University of Glasgow, Glasgow, UK; Centre for Immunobiology, School of Infection and Immunity, University of Glasgow, Glasgow, UK; Centre for Immunobiology, School of Infection and Immunity, University of Glasgow, Glasgow, UK; Centre for Immunobiology, School of Infection and Immunity, University of Glasgow, Glasgow, UK

**Keywords:** immune memory, interferon-gamma, influenza A virus, lung, trained immunity

## Abstract

Interferon gamma (IFN*γ*) is a potent antiviral cytokine that can be produced by many innate and adaptive immune cells during infection. Currently, our understanding of which cells produce IFN*γ* and where they are located at different stages of an infection is limited. We have used reporter mice to investigate *in vivo* expression of *Ifnγ* mRNA in the lung and secondary lymphoid organs during and following influenza A virus (IAV) infection. We observed a triphasic production of *Ifnγ* expression. Unconventional T cells and innate lymphoid cells, particularly NK cells, were the dominant producers of early *Ifnγ*, while CD4 and CD8 T cells were the main producers by day 10 post-infection. Following viral clearance, some memory CD4 and CD8 T cells continued to express *Ifnγ* in the lungs and draining lymph node. Interestingly, *Ifnγ* production by lymph node natural killer (NK), NKT, and innate lymphoid type 1 cells also continued to be above naïve levels, suggesting memory-like phenotypes for these cells. Analysis of the localization of *Ifnγ*+ memory CD4 and CD8 T cells demonstrated that cytokine+ T cells were located near airways and in the lung parenchyma. Following a second IAV challenge, lung IAV-specific CD8 T cells rapidly increased their expression of *Ifnγ* while CD4 T cells in the draining lymph node increased their *Ifnγ* response. Together, these data suggest that *Ifnγ* production fluctuates based on cellular source and location, both of which could impact subsequent immune responses.

## Graphical Abstract

**Figure:**
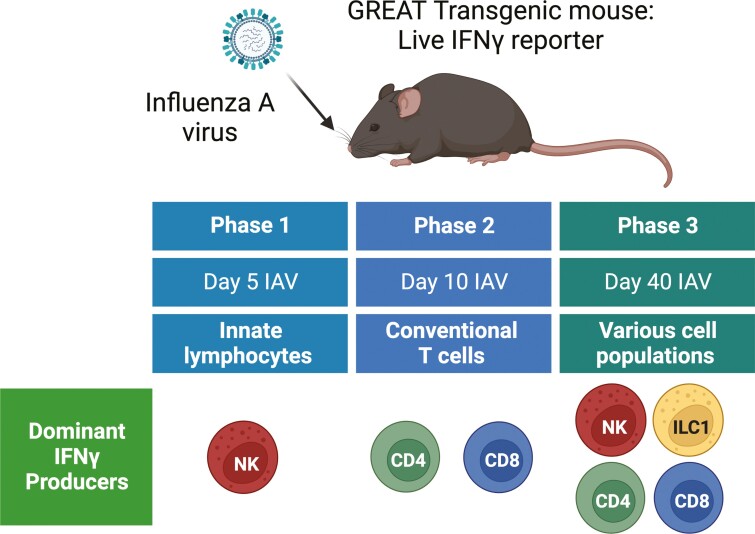

## Introduction

Interferon-gamma (IFN*γ*) is a key cytokine that plays multiple roles in the host immune response to viral infections, for example, influenza A virus (IAV). IFN*γ* promotes innate and adaptive leukocyte recruitment to the site of infection through the induction of CCR2 and CXCR3 ligands [1, 2]. Moreover, IFN*γ* signalling upregulates antigen presentation through major histocompatibility class I (MHCI) and MHCII pathways to CD8 and CD4 T cells, respectively [3–5], promoting T cell activation and ultimately facilitating viral clearance.

Various different immune cells have been documented to produce IFN*γ* following IAV infection. These include innate lymphoid cells (natural killer (NK) cells [6], innate lymphoid 1 cells (ILC1s [7])), unconventional T cells (NKT [8] and *γ*δ T cells [9, 10]) as well as classical αβ T cells (CD4 and CD8 T cells [11, 12]). However, the dynamics of when and how much IFN*γ* these cells can produce at different stages of IAV infection, and after re-infection, have not been comprehensively and simultaneously analysed.

IFN*γ* production by CD4 and CD8 T cells is correlated with protection from IAV infection in humans [13–16]. Most of these studies examine peripheral blood mononuclear cells, although IFN*γ*+ CD8 T cells increase in the bronchoalveolar lavage fluid during challenge infections [17]. Similar findings have been observed in mouse models of IAV infection demonstrating that IFN*γ*+ CD4 and CD8 T cells can protect mice from challenge infection [18–20]. A major advantage of mouse infection models is the ability to examine immune cells within different organs at multiple time points following infection. This can provide a broader understanding of the cell types that are key IFN*γ* producers following IAV infection. A further advantage of mouse studies is the ability to identify cytokine-producing cells via fluorescent reporter proteins [21, 22]. Thus, IFN*γ*+ cells can be detected without the need for *ex vivo* stimulation providing a more accurate view of the cells that respond to the virus *in vivo*.

Here, we characterised *Ifnγ* expression at different timepoints following IAV infection within the spleen, mediastinal lymph node (Med LN), and in the lungs of *Ifnγ* mRNA reporter mice [23]. As expected, innate cells, particularly NK cells, were the most prominent *Ifnγ* producers early post-infection, while conventional T cells were the largest *Ifnγ*+ population at day 10. In contrast, following IAV re-challenge, T cells rapidly produced *Ifnγ*, demonstrating their memory potential.

Interestingly, elevated *Ifnγ* levels were sustained at day 40 post-infection, even though IAV is cleared by day 10 [24, 25]. At this timepoint, CD4 and CD8 T cells were the only cells in the lungs that exhibited elevated *Ifnγ* expression. In contrast, raised *Ifnγ* expression at day 40 was found in ILC1s, NK, and NKT cells, as well as conventional T cells, in the Med LN. The continued heightened production of *Ifnγ* by non-adaptive immune cells echoes evidence that innate cells, including ILC1s [26, 27], NK [28], and NKT cells [29] can display memory-like properties and increased responsiveness to inflammatory stimuli.

Together, these data provide a comprehensive study of immune cell-derived *Ifnγ* expression over the course of IAV infection. This new in-depth understanding of *Ifnγ* expression from different cell types may facilitate the design of interventions that either boost or inhibit cytokine production.

## Materials and methods

### Study design

The aim of this study was to understand how *Ifnγ* expression by innate and adaptive immune cells changes over time following a viral infection. We used an influenza virus infection model in reporter mice and tracked responding memory T cells using MHCI and MHC II tetramers and the reporter systems in the GREATxSMART transgenic mice. A description of the experimental parameters, samples sizes, any samples that were excluded, and the statistical analysis are described in each figure legend. No specified randomization was conducted.

### Animals

Ten-week-old female C57BL/6 mice were purchased from Envigo (UK). C57BL/6 and GREATxSMART mice, original made by Richard Locksley [23, 30] and initially provided by David Withers, University of Birmingham, were maintained at the University of Glasgow under specific pathogen-free conditions in accordance with UK home office regulations (Project Licenses P2F28B003 and PP1902420) and approved by the local ethics committee. GREATxSMART mice have been described previously [30].

### Infections

IAV was prepared and titrated in MDCK cells. Mice were briefly anesthetized using inhaled isoflurane and infected with 200 plaque-forming units of IAV strain WSN in 20 μl of PBS intranasally (i.n.). Infected mice were rechallenged with 200 PFU of X31 (H3N2) where stated. Infected mice were weighed daily for 14 days post-infection. Any animals that lost more than 20% of their starting weight were humanely euthanized.

### Tissue preparation

Mice were injected intravenously (i.v.) with 1 μg anti-CD45-PE (ThermoFisher: clone: 30F11) 3 min before being euthanized by cervical dislocation. Spleen and mediastinal lymph nodes were processed by mechanical disruption. Single-cell suspensions of lungs were prepared by digestion with 1 mg/ml collagenase and 30 μg/ml DNAse (Sigma) for 40 min at 37°C in a shaking incubator. Red blood cells were lysed from spleen and lungs using lysis buffer (ThermoFisher).

### Flow cytometry staining

Single-cell suspensions were stained with PE or APC-labelled IA^b^/NP_311-325_ or APC-labelled D^b^/NP_368-374_ tetramers (NIH tetramer core) at 37°C, 5% CO_2_ for 2 h in complete RPMI (RPMI with 10% foetal calf serum, 100 μg/ml penicillin-streptomycin, and 2 mM l-glutamine) containing Fc block (24G2). Surface antibodies were added and the cells were incubated for a further 20 min at 4°C. Antibodies used were: anti-CD3 BV785 (BioLegend; clone: 17A2), anti-CD4 BUV805 (BD Bioscience; clone: RM4-5), anti-CD8 BV421 (ThermoFisher; clone: 53-6.7), anti-CD44 BUV395 (BD; clone: IM7), anti-CD45.2 BV605 (BioLegend; clone: 104), anti-CD69 PE-Cy7 (ThermoFisher; clone: H1.2F3), anti-CD127 APC (ThermoFisher; clone: A7R34), anti- *γ*δ TCR PE-Cy7 (BioLegend; clone: GL3), anti-ICOS BV785 (BioLegend; clone: C398.4A), anti-NK1.1 APC-Cy7 (BioLegend; clone: PK136), anti-PD1 BV711 (BioLegend; clone: 29F,1A12), and ‘dump’ antibodies: B220 (RA3-6B2), F4/80 (BM8), and MHC II (M5114) all on eFluor-450 (ThermoFisher) or PerCP-Cy5.5 (ThermoFisher; B220 and F4/80, and BioLegend; MHCII). Cells were stained with a fixable viability dye eFluor 506 (ThermoFisher). Stained cells were acquired on a BD LSR Fortessa and analysed using FlowJo (version 10, BD Bioscience).

### FACS

T cells were isolated from single-cell suspensions of mediastinal lymph nodes and lungs using a T cell isolation kit as per the manufacturer’s instructions (Stem Cell). Cells were stained with surface antibodies and sorted on a FACS Aria IIU. CD4+ or CD8+ TCRβ+CD44hi cells that were EYFP+ or EYFP negative were sorted into Qiagen RLT buffer and stored at –80°C.

### qPCR

RNA was extracted following the manufacturer’s instructions (Qiagen RNAeasy microkit) and analysed by qPCR (SYBR Green FastMix (Quanta Bioscience). *Ifnγ* and *18s* standards were generated from spleen cells from IAV-infected mice (Standard Primers: *Ifnγ*: Forward: ATCTGGAGGAACTGGCAAAA; Reverse: AGATACAACCCCGCAATCAC; *18s* Forward: CGTAGTTCCGACCATAAACGA; Reverse: ACATCTAAGGGCATCACAGACC) and purified by gel extraction (Quick Gel Extraction kit, Invitrogen). qPCR was performed on a QuantStudio 7 flex and expression was calculated using standard curves and results normalized to *18s* expression (qPCR Primers: *Ifnγ*: Forward: AGCAAGGCGAAAAAGGATG; Reverse: CTGGACCTGTGGGTTGTTG; *18s*: Forward: GACTCAACACGGGAAACCTC; Reverse: TAACCAGACAAATCGCTCCAC).

### Lung immunofluorescence imaging

Lungs from GREATxSMART mice were perfused with 5 mM EDTA and 1% PFA prior to removal and incubated at 4°C in 1% PFA and 30% sucrose for 24 h each, frozen in OCT and stored at –80°C. Ten micrometre lung sections were cut onto SuperFrost microscope slides (ThermoFisher) and stored at –20◦C prior to staining. Sections were incubated in Fc block (24G2) for 10 min to block non-specific binding, washed in 0.5% BSA/PBS (ThermoFisher), and stained with antibodies overnight. Antibodies used: anti-GFP (Life Technologies), anti-CD4 AF647 (BD; clone: RM4-5), anti-CD8b APC (BioLegend, clone; 53-5.8), anti-EpCAM AF594 (BioLegend: G8.8), and anti-MHCII eFluor450 (ThermoFisher; clone: M5114), slides were washed in 0.5% BSA/PBS and mounted using Vectashield mounting reagent (Vector Laboratories). Immunofluorescence images were acquired using a Zeiss LSM800 microscope, analysed using Volocity (version 7, Quorum Technologies), and example images were generated on Zen 2 lite.

### Statistical analysis

Data were analysed using Prism version 9 software (GraphPad). Groups were tested for normality using a Shapiro–Wilk test, and differences between groups were analysed by one-way ANOVAs, Kruskall–Wallis, or unpaired *t*-tests as indicated in figure legends. In all figures based on distribution normality, * represents a *P* value of <0.05; **P > 0.01, ***P > 0.001, ****P > 0.0001.

## Results

### Unconventional T cells and innate lymphoid cells produce *Ifnγ* in the early and adaptive immune phases in response to IAV infection in the lung and mediastinal lymph node

We used reporter mice, GREAT [23], to identify immune cells that express cytokines *in vivo* during and following an influenza A virus infection (IAV). These mice were crossed to IL-17 reporter mice, SMART [30], but in these experiments, we have focused on *Ifnγ*-producing cells. GREAT mice report live expression of *Ifnγ* mRNA via expression of EYFP [23], which can be detected by flow cytometry (gating shown in Supplementary Fig. 1). EYFP+ cells may not be IFN*γ* protein positive, particularly in resting T cells, which are known to express *Ifnγ* mRNA in the absence of protein [31]. We examined immune cells in the spleen, the lung draining, Med LN, and in the lung at early (day 5), peak adaptive immune response (day 10), and memory (day 40) timepoints [32].

Following IAV infection, we could identify multiple populations of *Ifnγ*+ immune cells (Supplementary Figs. 2 and 3). By injecting the animals with fluorescently labelled anti-CD45 shortly before euthanasia, we could distinguish cytokine-producing cells present in the blood from those in the tissues [33]. We have focused on the cells in the lung tissue rather than blood, as these are the cells that are most likely to contribute to viral control and clearance.

**Figure 1:**
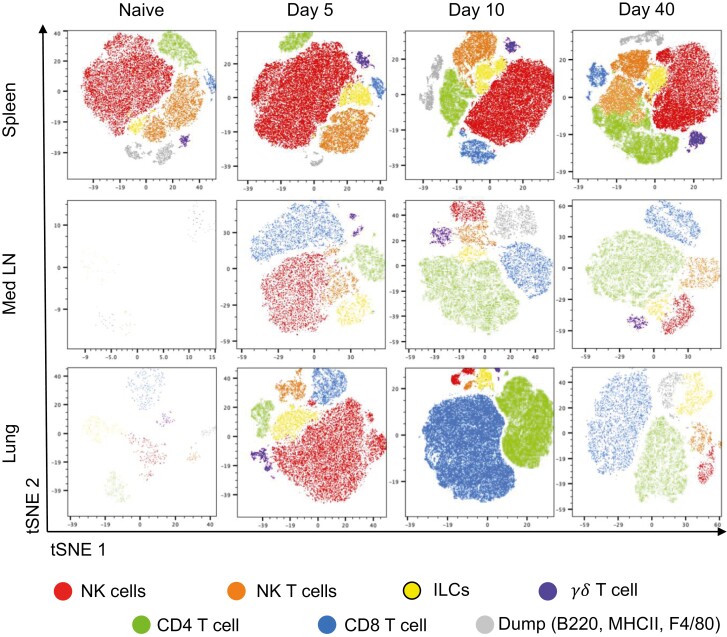
altered populations of *Ifnγ*+ immune cells at different time points following influenza virus infection. GREATXSMART mice were infected with IAV on day 0 and injected with fluorescently labelled anti-CD45 i.v. 3 min prior to removal of the spleen, Med LN, and lung at days 5, 10, or 40 post-infection. Single-cell suspension were analysed by flow cytometry. Representative tSNE plots depict CD45iv-negative *Ifnγ*/*EYFP*+ clusters within the spleen, Med LN, and lung were examined by flow cytometry at the indicated timepoints. Cell types identified as gated in Supplementary Fig. 1 as NK cells (red), NKT cells (orange), ILCs, (yellow), CD4 T cells (green), CD8 T cells (blue), *γ*δT cells (purple), and dump (B220, MHCII, and F4/80) channel cells (grey).

The majority of EYFP+ cells were cells known to produce IFN*γ* from previous studies: NK cells, NKT cells, ILC1s, *γ*δ T cells, CD4, and CD8 T cells [6–12] (Fig. 1). Smaller populations were positive for our ‘dump’ antibodies that included B220, MHCII, and F4/80, suggesting a small proportion of B cells and/or DCs and macrophages can produce *Ifnγ*. A small percentage (1–2%) of *Ifnγ*+ cells was not included in any of the gates shown in Supplementary Fig. 1.

**Figure 2:**
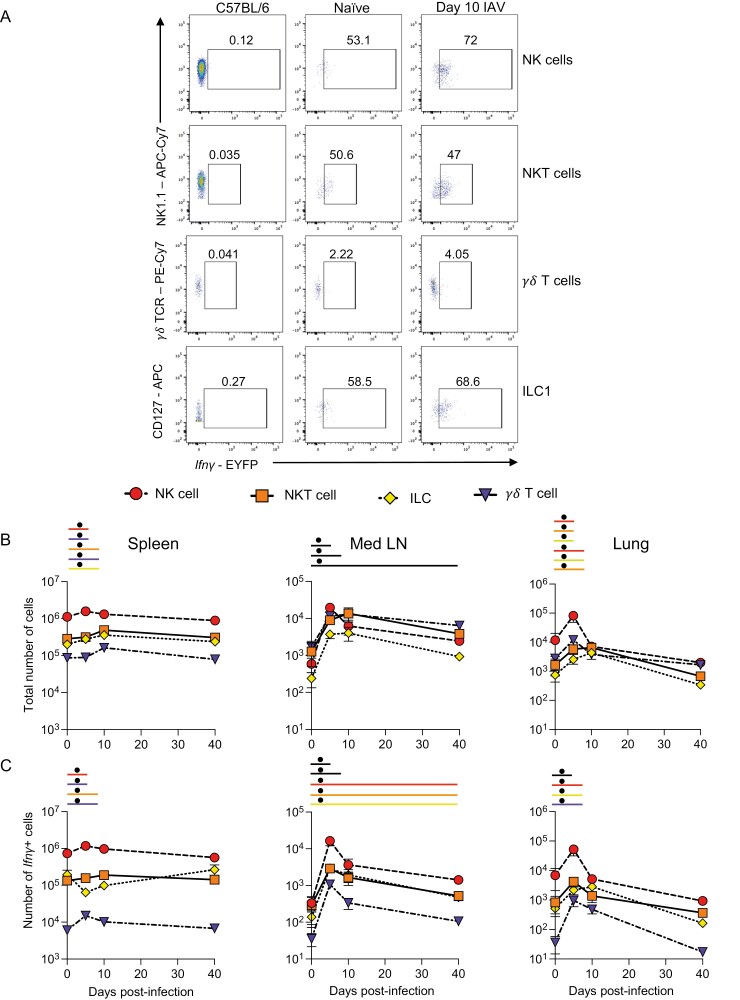
*Ifn* *γ* expression by unconventional T cells and innate lymphoid cells occurs early after IAV infection. GREATxSMART mice were infected with IAV on day 0 and injected with fluorescently labelled anti-CD45 i.v. 3 min prior to removal of the spleen, Med LN, and lung. Representative flow plots of EYFP+ NK, NK T, *γ*δT cells, and ILC1s at 10 days post-infection compared to C57BL/6 control (A). The total number (B) or number of *Ifnγ*+ cells (C) of the indicated cell populations were examined in naïve animals or those infected 5, 10, or 40 days previously. Each point represents the mean of 8–11 infected mice from 2 independent time course experiments; 21 naïve mice are combined from across the time points and experiments, error bars are SEM. Coloured lines above each timepoints relate to a significantly increased number of total cells or *Ifnγ*+ cells compared to naïve animals. If the line is black, this represents all cell types were higher at the indicated time point compared to naïve mice. Significance tested via a Kruskal–Wallis test followed by a Dunn’s multiple comparison test, • significant difference between indicated timepoint and naïve samples, refer to Supplementary Tables 1 and 2 for significance value.

To track the dynamic changes in the main *Ifnγ*+ populations during IAV infection, we first quantified the total numbers and numbers of *Ifnγ*+ innate and unconventional T cells over time (Fig. 2A–C, Supplementary Tables 1–and 2).

There were minor, but in some cases significant, changes in the numbers of NK, NKT, ILC1s, and *γ*δ T cells in the spleen following IAV infection (Fig. 2B). The numbers of *Ifnγ*+ NK, NKT, and *γ*δ T cells did increase significantly at either day 5 or day 10 post-infection but these changes were small (Fig. 2C). At all timepoints, NK cells were the largest population of splenic *Ifnγ*+ cells.

IAV infection led to more substantial changes in the total populations and numbers of *Ifnγ*+ cells in the Med LN. Here, there were significant increases in the numbers of NK cells, NKT cells, ILC1s, and *γ*δ T cells at all time points post-infection compared to naïve animals (Fig. 2B). These results were also reflected in the numbers of *Ifnγ*+ cells. *Ifnγ*+ cells from all populations were increased at days 5 and 10, and all but *γ*δ T cells remained increased at day 40 despite clearance of IAV by day 10 [24, 25] (Fig. 2C). These data suggest a sustained immune response to IAV and evidence for innate or trained memory. As in the spleen, NK cells were the largest population of *Ifnγ*+ cells within these cell types.

In the lung tissue, the numbers of ILC1s, NK, and NKT cells were increased 5 days after IAV infection compared to naïve animals (Fig. 2B). While the numbers of ILC1s and NKT cells remained elevated in the lung 10 days post-infection, all four cell types returned to naïve levels by day 40 post-infection.

The numbers of *Ifnγ*+ cells within each of the four populations were increased at day 5 post-infection and ILC1s and *γ*δ T cells remained elevated at day 10. Notably, the numbers of total NK cells and NKT cells dropped substantially by day 10 and were slightly lower than in naïve animals. In contrast to the sustained *Ifnγ* in the Med LN, the number of *Ifnγ*+ unconventional T cells and innate lymphoid cells returned to naïve levels by day 40 post-infection.

Together, these data suggest that of the innate cells, NK cells are the predominant source of *Ifnγ* in the spleen, Med LN, and lung. As expected, in the spleen and lung, the numbers of *Ifnγ*+ cells returned to naïve levels of *Ifnγ* by day 40 post-infection. In contrast, in the Med LN, we found evidence for long-term changes to several innate immune lymphocyte populations.

### CD4 and CD8 T cells are the predominant sources of *Ifnγ* after the clearance of IAV infection

**Figure 3:**
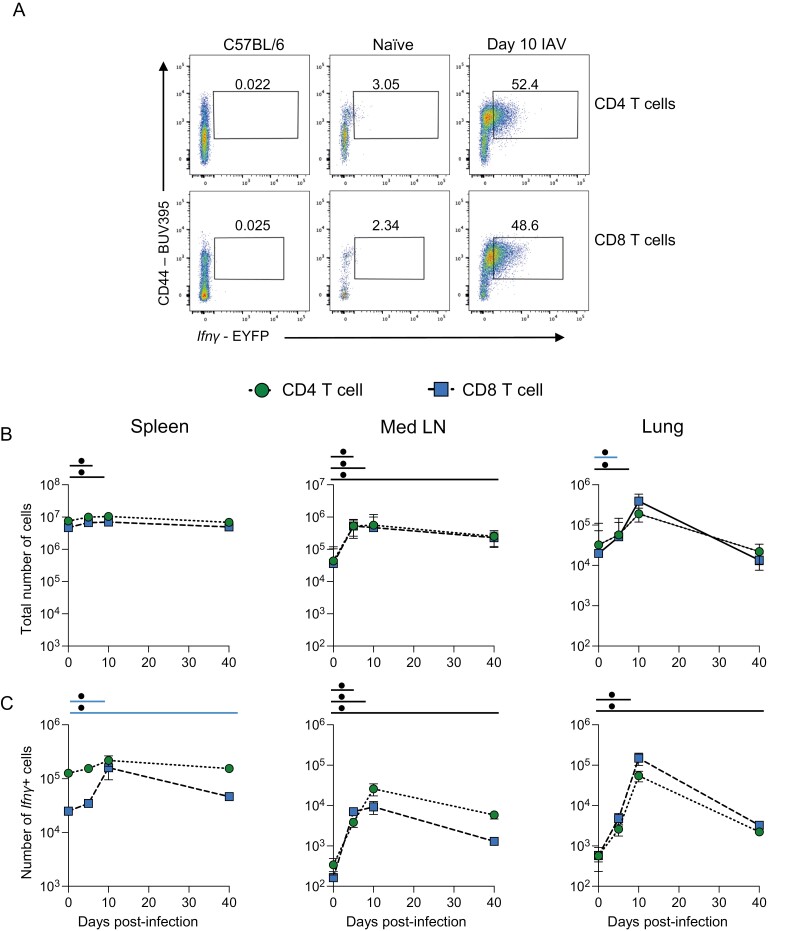
CD4 and CD8 T cells are the predominant source of *Ifnγ* after the clearance of IAV infection. GREATxSMART mice were infected with IAV on day 0 and injected with fluorescently labelled anti-CD45 i.v. 3 min prior to removal of the spleen, Med LN, and lung. Representative flow plots of EYFP+ CD4 and CD8 T cells at 10 days post-infection compared to C57BL/6 control (A). The total number (B) or number of *Ifnγ*+ cells (C) of the indicated cell populations were examined in naïve animals or those infected 5, 10, or 40 days previously. Each point represents the mean of 8–11 infected mice from two independent time course experiments; 21 naïve mice are combined from across the time points and experiments, error bars are SEM. Coloured lines above each timepoints relate to a significantly increased number of the total number of *Ifnγ*+ cells compared to naïve animals of a specific cell type. If the line is black, this represents both cell types were higher at the indicated time point compared to naïve mice. Significance tested via a Kruskal–Wallis test followed by a Dunn’s multiple comparison test, •significant difference between indicated timepoint and naïve samples, refer to Supplementary Tables 1 and 2 for significance value.

T cells are key IFN*γ*-producing populations during IAV infection [11, 12]. In the same experiments, therefore, we examined changes in *Ifnγ* expression by CD4 and CD8 T cells over the course of IAV infection (Fig. 3A). There were minor changes in the numbers of total CD4 and CD8 T cells in the spleen after IAV infection (Fig. 3B and Supplementary Tables 1 and 2). While the numbers of *Ifnγ*+ CD4 T cells remained stable, IAV infection did lead to a sustained increased in *Ifnγ*+ CD8 T cells up to 40 days post-infection (Fig. 3C).

In the Med LN, at all time points following infection, the numbers of total cells, and numbers of *Ifnγ*+, CD4, and CD8 T cells were increased compared to naïve animals (Fig. 3B and C). The numbers of *Ifnγ*+ T cells peaked at day 10 post-infection with similar numbers of cytokine+ CD4 and CD8 T cells. At day 40, their numbers remained above those in naïve animals (Fig. 3C).

Within the lung tissue, there were no increases in the numbers of *Ifnγ*+ CD4 and CD8 T cells 5 days after infection (Fig. 3C). By day 10, the numbers of total T cells and *Ifnγ*+ T cells increased substantially and then declined by day 40. In contrast to the innate and unconventional T cells, the numbers of *Ifnγ*+ CD4 and CD8 T cells did remain above naïve levels at day 40, indicating persistence of effector cytokine-producing memory T cells. Additionally, by comparing the numbers of *Ifnγ*+ cells from the different populations in the Med LN and lung at days 10 and 40 post-infection, we found that CD4 and CD8 T cells were the largest population of cytokine+ cells (Supplementary Fig. 3).

We also examined the mean fluorescence intensity (MFI) of the EYFP signal to determine the relative amounts of *Ifnγ* produced by the different lymphocytes across the time course (Supplementary Fig. 4). For most of the innate cell types, there were limited, but in some cases significant, changes in the EYFP MFI in the spleen. Interestingly, CD4 and CD8 T cells expressed low levels of *Ifnγ* at day 10 in the spleen and Med LN, suggesting these cells may receive a negative feedback signal at this time or the high cytokine+ cells may have migrated to the lung. At day 5 post-infection, in parallel with the increase in the numbers of *Ifnγ*+ ILC1, NK, and NKT cells in the Med LN and lung, these cells increased the amount of *Ifnγ* they produced. Similarly, lung CD4 and CD8 T cells increased their expression of *Ifnγ* at day 10 post-infection, matching their increase in cell number.

To confirm that the *Ifnγ* reporter marked cells still expressed *Ifnγ* transcript at the memory timepoints, we FACS sorted EYFP+ and negative CD44 high CD4 and CD8 T cells from the Med LN and lungs of IAV-infected mice. The EYFP+ CD4 T cells from these organs expressed more *Ifnγ* transcript than non-EYFP+ cells (Supplementary Fig. 5). In contrast, the CD8 EYFP+ cells expressed similar transcript levels to EYFP negative cells and the transcripts levels were lower than for the CD4 T cells. Potentially there may be a technical reason for this difference, including a loss of *Ifnγ* transcript *ex vivo* by the CD8 T cells. Alternatively, *Ifnγ* transcript may be more rapidly degraded by CD8 than CD4 T cells *in vivo.*

### EYFP+ CD4 and CD8 T cells are located near airways and in the lung parenchyma

**Figure 4:**
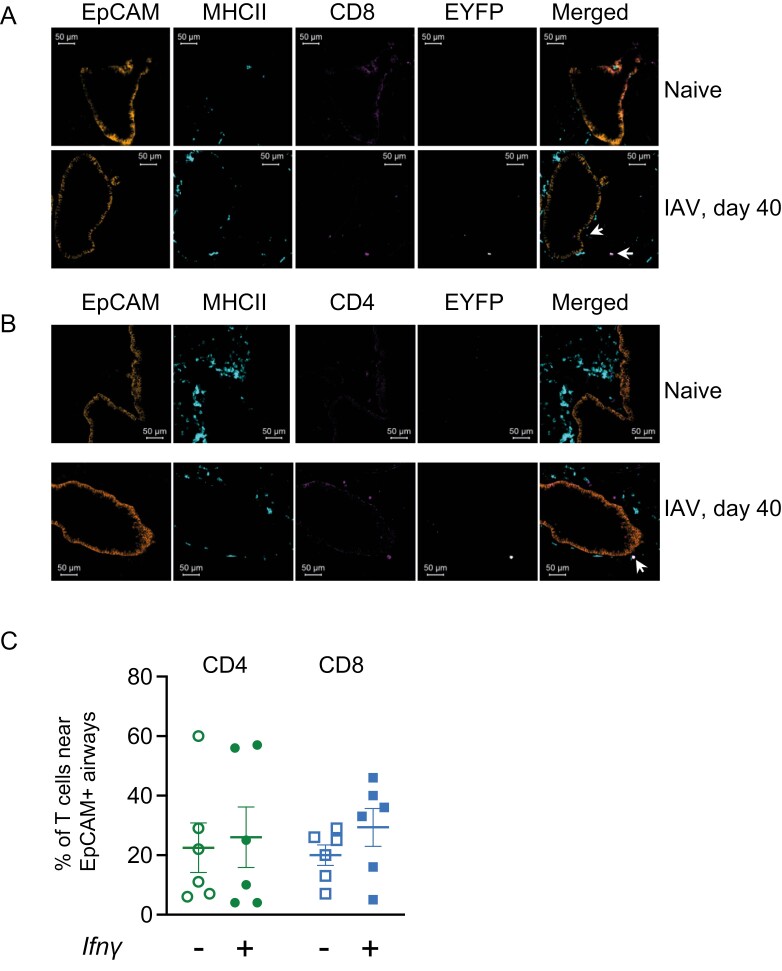
*EYFP+ CD4 and CD8 T cells are located near airways and in the lung parenchyma.* GREATxSMART mice were infected with IAV on day 0 and lungs were analysed after 40 days. Example images of lung EYFP (white), CD8 (A), and CD4 (B) (pink) cells are shown alongside EpCAM+ cells (orange) and MHCII+ cells (turquoise) in naïve and infected animals and the percentages of CD4 or CD8 T cells either in close proximity to EpCAM+ airways or within the parenchyma determined in infected mice. In C, each symbol represents one mouse, data combined from 3 to 4 slides per animal. Data are combined from two experiments with 3 mice per experiment. Scale bars are 50 μm and arrows show *Ifn*γ**+ CD4 or CD8 T cells.

We used immunofluorescence to investigate the location of the lung CD4 and CD8 T cells that continue to express *Ifnγ*. We hypothesized that these cells would be located near the airways, ready to respond to a subsequent infection. Both *Ifnγ*+ and negative CD4 and CD8 T cells were located both near EpCAM+ airways and in the parenchyma and this varied between animals (Fig. 4). These data suggest that during the maintenance of T cell memory, there is no specialized niche in which the *Ifnγ* cytokine+ T cells are located.

### IAV-specific CD4+ T cells continue to produce *Ifnγ* following viral clearance

**Figure 5:**
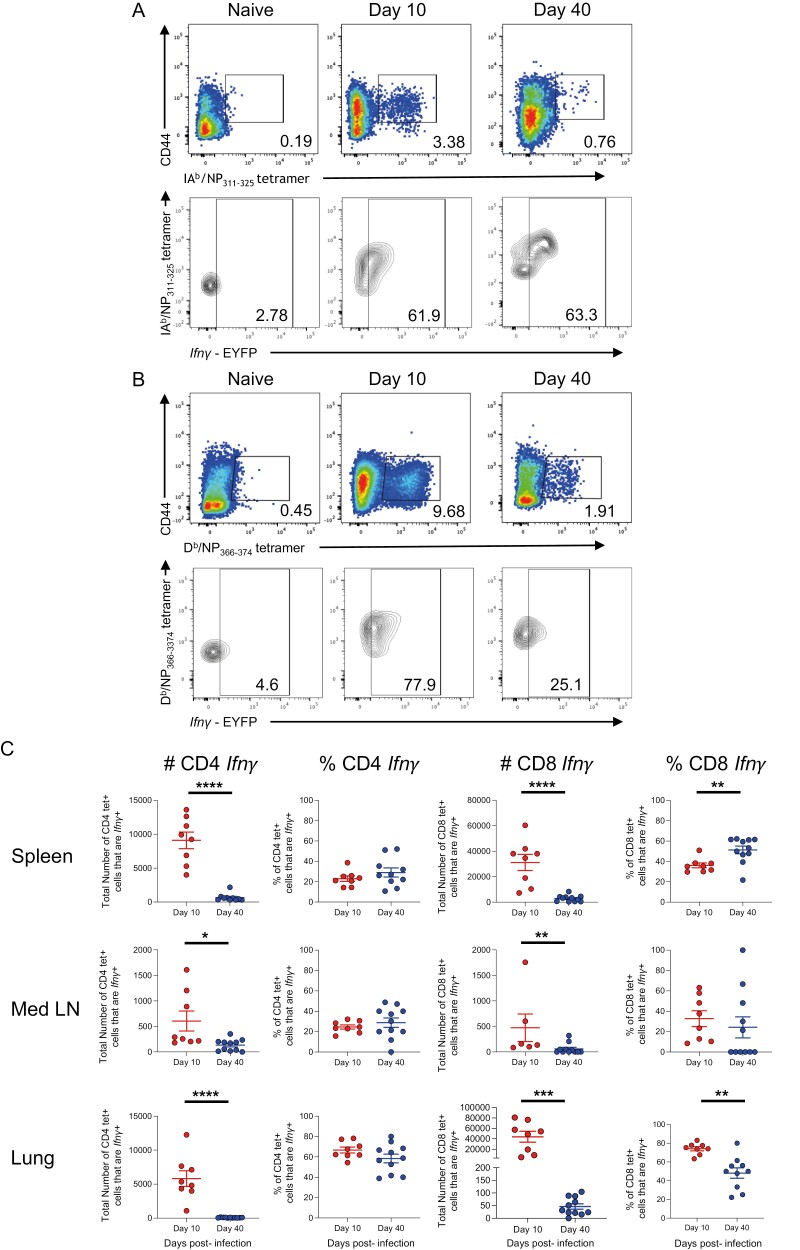
*IAV-specific memory CD4+ T cells maintain the ability to transcribe* Ifn**γ* after viral clearance.* GREATxSMART mice were infected with IAV on day 0 and injected with fluorescently labelled anti-CD45 i.v. 3 min prior to removal of the spleen, Med LN, and lung. Representative flow plots display staining of (A) IAV-specific *Ifn*γ**+ CD4 or (B) CD8 T cells from the lung. (C) The frequency (left) and total number (right) of IAV-specific CD4 and CD8 T cells were compared between the peak T cell response (day 10; red) and after viral clearance (day 40; blue). Each point represents an individual mouse from 8-11 mice combined from two independent experiments; error bars are SEM. Significance tested via an unpaired t test, *P < 0.05, **P < 0.01, ***P < 0.001, ****P < 0.0001.

To confirm that IAV-specific CD4 and CD8 T cells were *Ifnγ*+, we stained cells from IAV-infected reporter mice with MHC I and MHC II tetramers containing immunodominant IAV nucleoprotein (NP) peptides, NP_368-74_, and NP_311-325_, respectively at days 10 and 40 post-infection (Fig. 5A and B).

In the spleen, Med LN, and lung, the numbers of *Ifnγ*+ IA^b^/NP_311-325_ tetramer + CD4 and D^b^/NP_368-374_ tetramer+ CD8 T cells declined between the peak of the T cell response (day 10) and the memory time point (day 40) (Fig. 5C). This decline in T cell number coincides with the vast majority of effector T cells dying by apoptosis, and the formation of long-lived memory T cells [34, 35].

We also compared the percentages of CD4 and CD8 T cells that were *Ifnγ*+ between day 10 and day 40 (Fig. 5C). For IAV-specific CD4 T cells, there were no differences in any of the organs. This suggests cells that express *Ifnγ* are as likely as non-*Ifnγ*+ cells to enter the memory pool. Alternatively, memory CD4 T cells may fluctuate in their ability to express *Ifnγ* depending on their location. In the spleen, 29% (±5.5%), and Med LN, 29% (±5.8%), of the MHCII tetramer + cells were *Ifnγ*+. In contrast, 59% (±3.5%) of the MHCII tetramer+ cells were *Ifnγ*+ in the lung.

At day 10 post-infection, the majority of CD8 MHCI tetramer+ cells in the lung were *Ifnγ*+. This dropped by approximately half by day 40 suggesting that non-*Ifnγ*+ CD8 T cells are more likely to enter the memory pool. In contrast, in the spleen, there was a greater percentage of *Ifnγ*+ CD8 MHCI tetramer+ cells at day 40 than day 10, while in the Med LN, there were no differences between day 10 and 40. These data may suggest that entry into the memory pool for CD8 T cells may follow different rules depending on the cell’s migration ability, and/or reflect changes in memory cell function depending on location at the time of analysis.

### *Ifn* *γ*+ NK cells and ILCs rapidly increase in the lung following re-infection

We next examined the impact of a second IAV infection on *Ifnγ* producing immune cells.

Reporter mice were infected with WSN IAV (H1N1) and 30 days later some of these animals were re-infected with X31 (H3N2) and responding cells were analysed after a further 3 days. As these viruses have different surface proteins, neutralizing antibody cannot prevent infection. However, as internal IAV proteins are more conserved, T cell epitopes are shared between these viruses and these contribute to rapid immune protection to the re-challenge infection [36–39].

**Figure 6:**
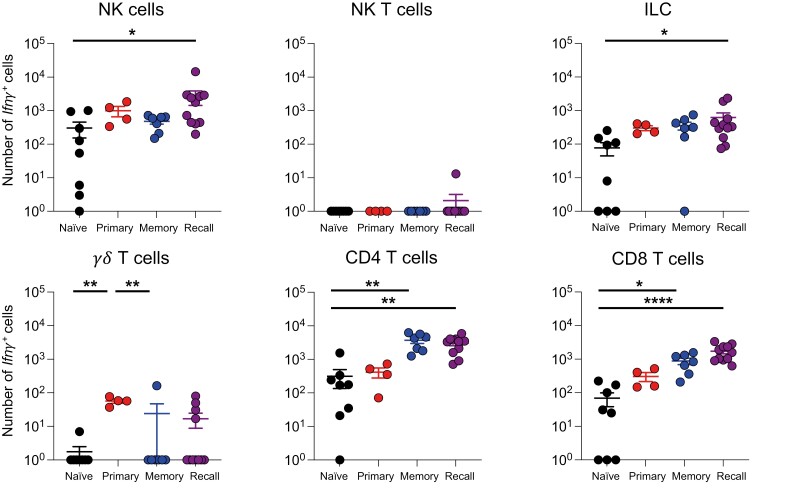
Ifn*γlung NK cells and ILC1s in the lung rapidly increase following challenge.* Naïve (primary) or WSN-infected GREATxSMART (recall) mice were infected i.n. with X31 for 3 days or left unchallenged (naïve and memory). The numbers of *Ifn*γ**+ cells of immune cells in lung were examined by flow cytometry. Each point represents an individual mouse from 4 to 11 mice combined from two independent experiments; error bars are SEM. Significance tested via Kruskal–Wallis test followed by a Dunn’s multiple comparison, *P < 0.05, **P < 0.01, ****P < 0.0001.

We focused on the innate and unconventional T cells in the lung as IFN*γ* is implicated in early IAV control [18, 40]. In comparison to naïve animals, we only found increased numbers of *Ifnγ*+ NK cells and ILCs in mice rechallenged with IAV, suggesting cytokine from these cells may be involved in early viral control (Fig. 6).

### IAV-specific CD4 and CD8 T cells are more activated and increase in *Ifnγ* expression following challenge infection

**Figure 7:**
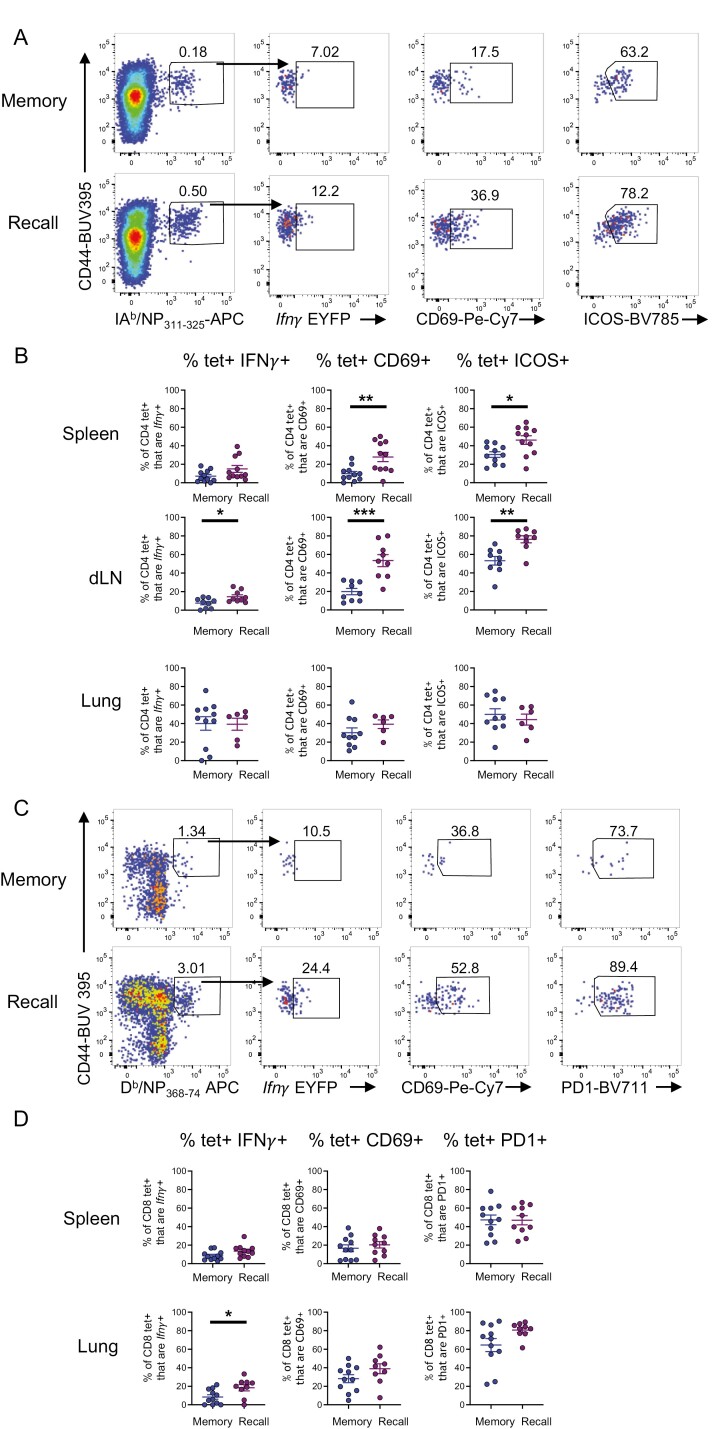
*IAV-specific CD4 and CD8 T cells are activated and increase* Ifn*γexpression following challenge infection.* GREATxSMART mice were either infected i.n. with IAV WSN (H1N1) for 40 days (memory) or re-infected with X31 (H3N2) for 3 days. Antigen-specific CD4 and CD8 T cells from the lung, spleen, and Med LN were examined. (A) Representative flow plots of IA^b^/NP_311-3250_-specific CD4+ T cells from the Med LN in memory (top) and recall infection (bottom) and expression of (B) *Ifn*γ**, CD69, and ICOS from IA^b^/NP_311-325_-specific CD4+ T cells in the spleen, Med LN, and lung. (C) Representative flow plots of D^b^/NP_368-374_-specific CD8+ T cells from the lung in memory (top) and recall infection (bottom) and expression of (D) *Ifn*γ**, CD69, and PD1 from D^b^/NP_368-374_-specific CD8+ T cells in the spleen and lung of memory and recall. Each point represents an individual mouse from 9 to 11 mice combined from two independent experiments; error bars are SEM. Significance tested via an unpaired *t* test, *P < 0.05, **P < 0.01, ***P < 0.001. Some samples were removed from these groups for technical reasons: too few MHC tetramer + cells, thymus contamination in the Med LN, or loss of cells during analysis.

While we did not observe changes in the numbers of *Ifnγ*+ CD4 and CD8 T cells between memory and re-infected animals (Fig. 6), by examining the antigen-specific T cell pools we found clear evidence for the re-activation of IAV-specific T cells (Fig. 7).

After re-infection, Med LN IAV-specific CD4 T cells increased their expression of *Ifnγ* and were more activated compared to IAV-specific CD4 T cells from memory mice based on increased expression of CD69 and ICOS (Fig. 7A and B). Splenic IAV-specific CD4 T cells were also activated by the re-infection, increasing their expression of CD69 and ICOS, although the expression of *Ifnγ* was equivalent to that in memory animals.

IAV-specific CD4 T cells in the lung did not display increased expression of CD69, ICOS, or *Ifnγ* following re-infection (Fig. 7B). However, we found that IAV-specific lung CD4 T cells were more likely to express these molecules than those in spleen or Med LN prior to infection. This suggests these cells remain in a semi-activated state following the initial infection.

We also compared the *Ifnγ* expression and activation state of D^b^/NP_368-374_ tetramer+CD8 T cells in the lung and spleen of memory and recall mice. There were no changes in splenic IAV-specific CD8 T cells and too few tetramer+ CD8 T cells in the Med LN for analysis. There was, however, a significant increase in *Ifnγ* expression from D^b^/NP_368-374_ tetramer+ CD8 T cells in the lungs following re-infection (Fig. 7C and D). These cells showed some evidence for increased expression of CD69 and PD1, although these changes were not significant.

## Discussion

Our data demonstrate triphasic *in vivo Ifnγ* expression following IAV infection. Unconventional T cells and innate lymphoid cells were early cytokine producers, prior to the expansion and infiltration of *Ifnγ*+ effector CD4 and CD8 T cells in secondary lymphoid organs and the lung. After viral clearance, *Ifnγ* levels remained above naïve levels in a number of cell types, including adaptive and innate immune cells, for at least 40 days.

We examined *Ifnγ* expression at four different time points following infection. Our data identified different populations as the largest producers in the antiviral response at different times. This was linked with the expansion of first innate and then adaptive cells in the lung and draining lymph nodes during the infection. We also examined cells at a late memory time point, and at day 3 post a re-challenge infection. Very early *Ifnγ* produced by memory CD8 T cells and NK cells has been observed in previously infected animals following a repeat infection with the same strain of IAV [41]. In our studies, we have used two different strains of IAV that do not share viral surface proteins and, therefore, cannot be controlled by neutralizing antibody. In such heterologous IAV infections, enhanced viral control is mainly mediated by T cells that recognize more conserved internal viral proteins and in mice is observed from day 3 post-infection onwards [36–39]. Therefore, we focused on a timepoint at which it is likely that the memory T cells would be acting to reduce the virus.

Our finding that NK cells provide early *Ifnγ* expression agrees with studies from Stegemann-Koniszewski and Wang [6, 42]. We have extended these data by demonstrating that NK cells are the dominant producers of *Ifnγ* during early IAV infection, both in terms of numbers of cells and amount of cytokine produced. We also provide evidence that NKT cells, ILC1s, and *γ*δ T cells also produce *Ifnγ* early during infection as other studies have described [7–10]. As expected, large numbers of CD4 and CD8 T cells produced *Ifnγ* at day 10 post infection [32, 43]. Unexpectedly, we found that a number of cell types continued to express *Ifnγ* at levels above naïve animals for at least 40 days. In the Med LN, these included CD4 and CD8 αβT cells, NKT cells, NK cells, and ILC1s. In all cases, the total numbers of these cells were also higher at day 40 post-IAV than in naïve animals suggesting either continued recruitment to or retention within the lymph node.

While long-term alterations to conventional T cells following viral challenge are to be expected, classically, unconventional T cells and innate immune cells do not display memory characteristics. This view has been challenged in the last 10–20 years, in particular in the fields of NK cells and monocytes [44, 45]. In IAV infection studies, Li *et al.* identified protective NK cells in the liver, but not the lung, of infected animals [46]. In contrast, Zheng *et al.* describe an alteration to splenic NK cells following IAV infection, leading to reduced cytotoxicity and an inability to protect adoptive immunodeficient hosts [47]. We found a rapid recruitment of cytokine + cells to the lungs following a secondary infection. The response may be a result of the increased *Ifnγ* + NK cells in the lymph node after the initial infection. Alternatively, the lung CD4 and CD8 T cell response to re-infection could lead to enhanced migration of NK cells to the lung. Such collaboration between different types of immune cells would demonstrate a coordinated response that would be difficult to unpick using traditional single adoptive transfer approaches.

The *Ifnγ*+ lung CD4 and CD8 T cells were negative for the CD45 antibody injected prior to analysis demonstrating that these cells are within the lung tissue at this time. At least a proportion of these cells may be tissue-resident memory (Trm) cells, in particular the CD69+ cells, although CD69 is not found in all Trms, especially CD4 Trm cells [48, 49]. CD4 and CD8 Trm T cells can protect against re-infection with IAV, including via IFN*γ* production [20, 50, 51]. The high baseline level of *Ifnγ* in the CD4 T cells and the rapid increase in expression by CD8 T cells suggest ongoing and new cytokine production may both contribute to this protection.

An alternative explanation for the continued elevated expression of *Ifnγ* by multiple cell types is that persistent IAV antigens fuel a low-level immune response. Studies from Jelly-Gibbs *et al.* and Zammit *et al.* demonstrate the presentation of IAV antigens to CD4 and CD8 T cells respectively for 1–2 months post infection [52, 53]. The continued presentation to conventional T cells could drive *Ifnγ* expression which in turn supports the cytokine production by the unconventional and innate T cells, for example, via promoting IL-12 production [54].

Zammit *et al.* tracked the persistent IAV antigen to the Med LN, but we found that T cells in the lung also continued to express *Ifnγ* to day 40 post-infection. Antigen could also be retained in the lung, for example, within clusters of immune cells [41, 55]. Various APC populations have been implicated in presenting antigen to tissue-resident memory cells including different dendritic cell populations [56, 57], upper and lower airway epithelial cells [58, 59], and lung fibroblasts [60]. From our imaging data, we found that *Ifnγ*+ memory CD4 and CD8 T cells were located near EpCAM+ airways and in the parenchyma. This perhaps suggests that if persistent antigen drives this *Ifnγ* response, a number of different cell types could drive this response.

In summary, our data demonstrate triphasic production of *in vivo Ifnγ* production, started by unconventional T cells and innate lymphoid cells early in infection, followed by effector T cells. IAV infection led to a classical T cell memory response in the lung, but also increases in *Ifnγ*+ ILC1, NK, and NKT cells in the draining lymph node, suggesting memory-like phenotypes. These novel findings identify different sources and localization of *Ifnγ* following viral infection that may impact on subsequent infections and improve our understanding of the generation of both classical and non-classical immune memory.

## Supplementary Material

kyad014_suppl_Supplementary_Material

## Data Availability

Data are available on request to the corresponding authors. ***Permission to reproduce***: The manuscript is published under CC-BY-NC licence. ***Animal research***: This study adheres to ARRIVE guidelines.

## Abbreviations

APC: antigen presenting cell
BSA: bovine serum albumin
CCR: C-C chemokine receptor
CD: cluster of differentiation
CXCR: C-X-C chemokine receptor
EDTA: ethylenediaminetetraacetic acid
EpCAM: epithelial cellular adhesion molecule
EYFP: enhanced yellow fluorescent protein
GREAT: IFN*γ* reporter with endogenous polyA tail
IAV: influenza A virus
ICOS: inducible T cell co-stimulator
IFN*γ*: interferon gamma
i.n.: intranasal
IL: interleukin
ILC: innate lympjoid cell
i.v.: intravenous
Med LN: mediastinal lymph node
MFI: mean florescence intensity
MHC: major histocompatibility complex
mRNA: messenger ribonucleic acid
NK: natural killer
NP: nucleoprotein
PBS: phosphate buffer saline
PD1: programmed cell death protein 1
PFA: paraformaldehyde
PFU: plaque-forming unit
SMART: surface marker for transcription
Trm: tissue-resident memory
qPCR: quantitative polymerase chain reaction

## References

[1] Rauch I , Müller M, Decker T. The regulation of inflammation by interferons and their STATs. JAK-STAT 2013, 2, e23820. doi:10.4161/jkst.23820. 24058799 PMC3670275

[2] Metzemaekers M , Vanheule V, Janssens R, Struyf S, Proost P. Overview of the mechanisms that may contribute to the non-redundant activities of interferon-inducible CXC chemokine receptor 3 ligands. Front Immunol 2018, 8, 1970. doi:10.3389/fimmu.2017.01970. 29379506 PMC5775283

[3] Hisamatsu H , Shimbara N, Saito Y, Kristensen P, Hendil KB, Fujiwara T, et al. Newly identified pair of proteasomal subunits regulated reciprocally by interferon gamma. J Exp Med 1996, 183, 1807–16. doi:10.1084/jem.183.4.1807. 8666937 PMC2192534

[4] Shirayoshi Y , Burke PA, Appella E, Ozato K. Interferon-induced transcription of a major histocompatibility class I gene accompanies binding of inducible nuclear factors to the interferon consensus sequence. Proc Natl Acad Sci USA 1988, 85, 5884–8. doi:10.1073/pnas.85.16.5884. 2457903 PMC281869

[5] Mach B , Steimle V, Martinez-Soria E, Reith W. Regulation of MHC class II genes: lessons from a disease. Annu Rev Immunol 1996, 14, 301–31. doi:10.1146/annurev.immunol.14.1.301. 8717517

[6] Stegemann-Koniszewski S , Behrens S, Boehme JD, Hochnadel I, Riese P, Guzmán CA, et al. Respiratory influenza A virus infection triggers local and systemic natural killer cell activation via toll-like receptor 7. Front Immunol 2018, 9, 245. doi:10.3389/fimmu.2018.00245. 29497422 PMC5819576

[7] Weizman OE , Adams NM, Schuster IS, Krishna C, Pritykin Y, Lau C, et al. ILC1 confer early host protection at initial sites of viral infection. Cell 2017, 171, 795–808.e12. doi:10.1016/j.cell.2017.09.052. 29056343 PMC5687850

[8] Maazi H , Singh AK, Speak AO, Lombardi V, Lam J, Khoo B, et al. Lack of PD-L1 expression by iNKT cells improves the course of influenza A infection. PLoS One 2013, 8, e59599. doi:10.1371/journal.pone.0059599. 23555047 PMC3598698

[9] Qin G , Liu Y, Zheng J, Ng IH, Xiang Z, Lam KT, et al. Type 1 responses of human V*γ*9Vδ2 T cells to influenza A viruses. J Virol 2011, 85, 10109–16. 21752902 10.1128/JVI.05341-11 PMC3196408

[10] Sant S , Jenkins MR, Dash P, Watson KA, Wang Z, Pizzolla A, et al. Human *γ*δ T-cell receptor repertoire is shaped by influenza viruses, age and tissue compartmentalisation. Clin Transl Immunol 2019, 8, e1079. doi:10.1002/cti2.1079. PMC6756999 31559018

[11] Hufford MM , Kim TS, Sun J, Braciale TJ. The effector T cell response to Influenza infection. Curr Top Microbiol Immunol 2015, 386, 423–55. doi:10.1007/82_2014_397. 25033753 PMC4224975

[12] Zens KD , Farber DL. Memory CD4 T cells in influenza. Curr Top Microbiol Immunol 2015, 386, 399–421. doi:10.1007/82_2014_401. 25005927 PMC4339101

[13] Tsang TK , Lam KT, Liu Y, Fang VJ, Mu X, Leung NHL, et al. Investigation of CD4 and CD8 T cell-mediated protection against influenza A virus in a cohort study. BMC Med 2022, 20, 230. 35858844 10.1186/s12916-022-02429-7 PMC9301821

[14] Wilkinson TM , Li CK, Chui CS, Huang AK, Perkins M, Liebner JC, et al. Preexisting influenza-specific CD4+ T cells correlate with disease protection against influenza challenge in humans. Nat Med 2012, 18, 274–80. doi:10.1038/nm.2612. 22286307

[15] Sridhar S , Begom S, Bermingham A, Hoschler K, Adamson W, Carman W, et al. Cellular immune correlates of protection against symptomatic pandemic influenza. Nat Med 2013, 19, 1305–12. doi:10.1038/nm.3350. 24056771

[16] Hayward AC , Wang L, Goonetilleke N, Fragaszy EB, Bermingham A, Copas A, et al.; Flu Watch Group. Natural T cell-mediated protection against seasonal and pandemic influenza. Results of the flu watch cohort study. Am J Respir Crit Care Med 2015, 191, 1422–31. doi:10.1164/rccm.201411-1988OC. 25844934 PMC4476562

[17] Paterson S , Kar S, Ung SK, Gardener Z, Bergstrom E, Ascough S, et al. Innate-like gene expression of lung-resident memory CD8+ T cells during experimental human influenza: a clinical study. Am J Respir Crit Care Med 2021, 204, 826–41. doi:10.1164/rccm.202103-0620OC. 34256007 PMC8528532

[18] Bot A , Bot S, Bona CA. Protective role of gamma interferon during the recall response to influenza virus. J Virol 1998, 72, 6637–45. doi:10.1128/JVI.72.8.6637-6645.1998. 9658110 PMC109853

[19] Strutt TM , McKinstry KK, Dibble JP, Winchell C, Kuang Y, Curtis JD, et al. Memory CD4+ T cells induce innate responses independently of pathogen. Nat Med 2010, 16, 558–64, 1p following 564. doi:10.1038/nm.2142. 20436484 PMC2927232

[20] Teijaro JR , Verhoeven D, Page CA, Turner D, Farber DL. Memory CD4 T cells direct protective responses to influenza virus in the lungs through helper-independent mechanisms. J Virol 2010, 84, 9217–26. doi:10.1128/JVI.01069-10. 20592069 PMC2937635

[21] Sarkar S , Heise MT. Mouse models as resources for studying infectious diseases. Clin Ther 2019, 41, 1912–22. doi:10.1016/j.clinthera.2019.08.010. 31540729 PMC7112552

[22] Croxford AL , Buch T. Cytokine reporter mice in immunological research: perspectives and lessons learned. Immunology 2011, 132, 1–8. doi:10.1111/j.1365-2567.2010.03372.x. 21070235 PMC3015069

[23] Reinhardt RL , Liang HE, Locksley RM. Cytokine-secreting follicular T cells shape the antibody repertoire. Nat Immunol 2009, 10, 385–93. doi:10.1038/ni.1715. 19252490 PMC2714053

[24] Zhao X , Lin X, Li P, Chen Z, Zhang C, Manicassamy B, et al. Expanding the tolerance of segmented Influenza A virus genome using a balance compensation strategy. PLoS Pathog 2022, 18, e1010756. doi:10.1371/journal.ppat.1010756. 35926068 PMC9380948

[25] Kim JH , Bryant H, Fiedler E, Cao T, Rayner JO. Real-time tracking of bioluminescent influenza A virus infection in mice. Sci Rep 2022, 12, 3152. 35210462 10.1038/s41598-022-06667-w PMC8873407

[26] Orr-El W , Eric S, Nicholas MA, Andrew DH, Luke R, Chirag K, et al. Mouse cytomegalovirus-experienced ILC1s acquire a memory response dependent on the viral glycoprotein m12. Nat Immunol 2019, 20, 1004–11. 31263280 10.1038/s41590-019-0430-1 PMC6697419

[27] Wang X , Peng H, Cong J, Wang X, Lian Z, Wei H, et al. Memory formation and long-term maintenance of IL-7Rα + ILC1s via a lymph node-liver axis. Nat Commun 2018, 9, 4854. 30451860 10.1038/s41467-018-07405-5 PMC6242895

[28] Rasid O , Chevalier C, Camarasa TM, Fitting C, Cavaillon JM, Hamon MA. H3K4me1 supports memory-like NK cells induced by systemic inflammation. Cell Rep 2019, 29, 3933–3945.e3. doi:10.1016/j.celrep.2019.11.043. 31851924

[29] Shimizu K , Sato Y, Shinga J, Watanabe T, Endo T, Asakura M, et al. KLRG+ invariant natural killer T cells are long-lived effectors. Proc Natl Acad Sci USA 2014, 111, 12474–9. doi:10.1073/pnas.1406240111. 25118276 PMC4151776

[30] Price AE , Reinhardt RL, Liang HE, Locksley RM. Marking and quantifying IL-17A-producing cells in vivo. PLoS One 2012, 7, e39750. doi:10.1371/journal.pone.0039750. 22768117 PMC3387253

[31] Salerno F , Engels S, van den Biggelaar M, van Alphen FPJ, Guislain A, Zhao W, et al. Translational repression of pre-formed cytokine-encoding mRNA prevents chronic activation of memory T cells. Nat Immunol 2018, 19, 828–37. doi:10.1038/s41590-018-0155-6. 29988089 PMC6643272

[32] Hornick EE , Zacharias ZR, Legge KL. Kinetics and phenotype of the CD4 T cell response to influenza virus infections. Front Immunol 2019, 10, 2351. doi:10.3389/fimmu.2019.02351. 31632414 PMC6783515

[33] Anderson KG , Mayer-Barber K, Sung H, Beura L, James BR, Taylor JJ, et al. Intravascular staining for discrimination of vascular and tissue leukocytes. Nat Protocols 2014, 9, 209–22. doi:10.1038/nprot.2014.005. 24385150 PMC4428344

[34] Sallusto F , Lanzavecchia A, Araki K, Ahmed R. From vaccines to memory and back. Immunity 2010, 33, 451–63. doi:10.1016/j.immuni.2010.10.008. 21029957 PMC3760154

[35] Kaech SM , Wherry EJ, Ahmed R. Effector and memory T-cell differentiation: implications for vaccine development. Nat Rev Immunol 2002, 2, 251–62. doi:10.1038/nri778. 12001996

[36] Liang S , Mozdzanowska K, Palladino G, Gerhard W. Heterosubtypic immunity to influenza type A virus in mice: effector mechanisms and their longevity. J Immunol (Baltimore, Md.: 1950) 1994, 152, 1653–61. 8120375

[37] Guo H , Baker SF, Martínez-Sobrido L, Topham DJ. Induction of CD8 T cell heterologous protection by a single dose of single-cycle infectious influenza virus. J Virol 2014, 88, 12006–16. doi:10.1128/JVI.01847-14. 25100831 PMC4178714

[38] Goplen NP , Wu Y, Son YM, Li C, Wang Z, Cheon IS, et al. Tissue-resident CD8+ T cells drive age-associated chronic lung sequelae after viral pneumonia. Sci Immunol 2020, 5, eabc4557. doi:10.1126/sciimmunol.abc4557. 33158975 PMC7970412

[39] Slütter B , Van Braeckel-Budimir N, Abboud G, Varga SM, Salek-Ardakani S, Harty JT. Dynamics of influenza-induced lung-resident memory T cells underlie waning heterosubtypic immunity. Sci Immunol 2017, 2, eaag2031. doi:10.1126/sciimmunol.aag2031. 28783666 PMC5590757

[40] Weiss ID , Wald O, Wald H, Beider K, Abraham M, Galun E, et al. IFN-gamma treatment at early stages of influenza virus infection protects mice from death in a NK cell-dependent manner. JInterferon Cytokine Res 2010, 30, 439–49. doi:10.1089/jir.2009.0084. 20235626

[41] MacLean AJ , Richmond N, Koneva L, Attar M, Medina CAP, Thornton EE, et al. TI Arnon: secondary influenza challenge triggers resident memory B cell migration and rapid relocation to boost antibody secretion at infected sites. Immunity 2022, 55, 718–733.e8. doi:10.1016/j.immuni.2022.03.003. 35349789 PMC9044924

[42] Wang J , Li F, Zheng M, Sun R, Wei H, Tian Z. Lung natural killer cells in mice: phenotype and response to respiratory infection. Immunology 2012, 137, 37–47. doi:10.1111/j.1365-2567.2012.03607.x. 22612500 PMC3449245

[43] Hufford MM , Kim TS, Sun J, Braciale TJ. Antiviral CD8+ T cell effector activities in situ are regulated by target cell type. J Exp Med 2011, 208, 167–80. doi:10.1084/jem.20101850. 21187318 PMC3023137

[44] Netea MG , Domínguez-Andrés J, Barreiro LB, Chavakis T, Divangahi M, Fuchs E, et al. Defining trained immunity and its role in health and disease. Nat Rev Immunol 2020, 20, 375–88. doi:10.1038/s41577-020-0285-6. 32132681 PMC7186935

[45] Ordovas-Montanes J , Beyaz S, Rakoff-Nahoum S, Shalek AK. Distribution and storage of inflammatory memory in barrier tissues. Nat Rev Immunol 2020, 20, 308–20. doi:10.1038/s41577-019-0263-z. 32015472 PMC7547402

[46] Li T , Wang J, Wang Y, Chen Y, Wei H, Sun R, et al. Respiratory influenza virus infection induces memory-like liver NK cells in mice. J Immunol (Baltimore, Md.: 1950) 2017, 198, 1242–52. doi:10.4049/jimmunol.1502186. 28031334

[47] Zheng J , Wen L, Yen HL, Liu M, Liu Y, Teng O, et al. Phenotypic and functional characteristics of a novel influenza virus hemagglutinin-specific memory NK cell. J Virol 2021, 95, 12. 10.1128/JVI.00165-21 PMC8316001 33827945

[48] Tang J , Sun J. Lung tissue-resident memory T cells: the gatekeeper to respiratory viral (re)-infection. Curr Opin Immunol 2023, 80, 102278. doi:10.1016/j.coi.2022.102278. 36565508 PMC9911367

[49] Ugur M , Schulz O, Menon MB, Krueger A, Pabst O. Resident CD4+ T cells accumulate in lymphoid organs after prolonged antigen exposure. Nat Commun 2014, 5, 4821. doi:10.1038/ncomms5821. 25189091

[50] McMaster SR , Wilson JJ, Wang H, Kohlmeier JE. Airway-resident memory CD8 T cells provide antigen-specific protection against respiratory virus challenge through rapid IFN-*γ* production. J Immunol (Baltimore, Md.: 1950) 2015, 195, 203–9. doi:10.4049/jimmunol.1402975. PMC4475417 26026054

[51] Teijaro JR , Turner D, Pham Q, Wherry EJ, Lefrançois L, Farber DL. Cutting edge: tissue-retentive lung memory CD4 T cells mediate optimal protection to respiratory virus infection. J Immunol (Baltimore, Md.: 1950) 2011, 187, 5510–4. doi:10.4049/jimmunol.1102243. PMC3221837 22058417

[52] Jelley-Gibbs DM , Brown DM, Dibble JP, Haynes L, Eaton SM, Swain SL. Unexpected prolonged presentation of influenza antigens promotes CD4 T cell memory generation. J Exp Med 2005, 202, 697–706. doi:10.1084/jem.20050227. 16147980 PMC2212871

[53] Zammit DJ , Turner DL, Klonowski KD, Lefrançois L, Cauley LS. Residual antigen presentation after influenza virus infection affects CD8 T cell activation and migration. Immunity 2006, 24, 439–49. doi:10.1016/j.immuni.2006.01.015. 16618602 PMC2861289

[54] Yang YF , Tomura M, Ono S, Hamaoka T, Fujiwara H. Requirement for IFN-gamma in IL-12 production induced by collaboration between v(alpha)14(+) NKT cells and antigen-presenting cells. Int Immunol 2000, 12, 1669–75. doi:10.1093/intimm/12.12.1669. 11099306

[55] Turner DL , Bickham KL, Thome JJ, Kim CY, D’Ovidio F, Wherry EJ, et al. Lung niches for the generation and maintenance of tissue-resident memory T cells. Mucosal Immunol 2014, 7, 501–10. doi:10.1038/mi.2013.67. 24064670 PMC3965651

[56] Low JS , Farsakoglu Y, Amezcua Vesely MC, Sefik E, Kelly JB, Harman CCD, et al. Tissue-resident memory T cell reactivation by diverse antigen-presenting cells imparts distinct functional responses. J Exp Med 2020, 217, e20192291. doi:10.1084/jem.20192291. 32525985 PMC7398161

[57] Zammit DJ , Cauley LS, Pham QM, Lefrançois L. Dendritic cells maximize the memory CD8 T cell response to infection. Immunity 2005, 22, 561–70. doi:10.1016/j.immuni.2005.03.005. 15894274 PMC2857562

[58] Shenoy AT , Wasserman GA, Arafa EI, Wooten AK, Smith NMS, Martin IMC, et al. Lung CD4 + resident memory T cells remodel epithelial responses to accelerate neutrophil recruitment during pneumonia. Mucosal Immunol 2020, 13, 334–43. doi:10.1038/s41385-019-0229-2. 31748706 PMC7044037

[59] Toulmin SA , Bhadiadra C, Paris AJ, Lin JH, Katzen J, Basil MC, et al. Type II alveolar cell MHCII improves respiratory viral disease outcomes while exhibiting limited antigen presentation. Nat Commun 2021, 12, 3993. doi:10.1038/s41467-021-23619-6. 34183650 PMC8239023

[60] Kerdidani D , Aerakis E, Verrou KM, Angelidis I, Douka K, Maniou MA, et al. Lung tumor MHCII immunity depends on in situ antigen presentation by fibroblasts. J Exp Med 2022, 219, e20210815. doi:10.1084/jem.20210815. 35029648 PMC8764966

