# Supplementary material for: Triphasic production of IFNγ by innate and adaptive lymphocytes following influenza A virus infection
Source: Discov Immunol. 2023 Aug 19;2(1):kyad014. doi: 10.1093/discim/kyad014 (PMC10568397; doi:10.1093/discim/kyad014)
Supplement: kyad014_suppl_Supplementary_Material [file kyad014_suppl_Supplementary_Material.pdf]

Supplementary Figure 1

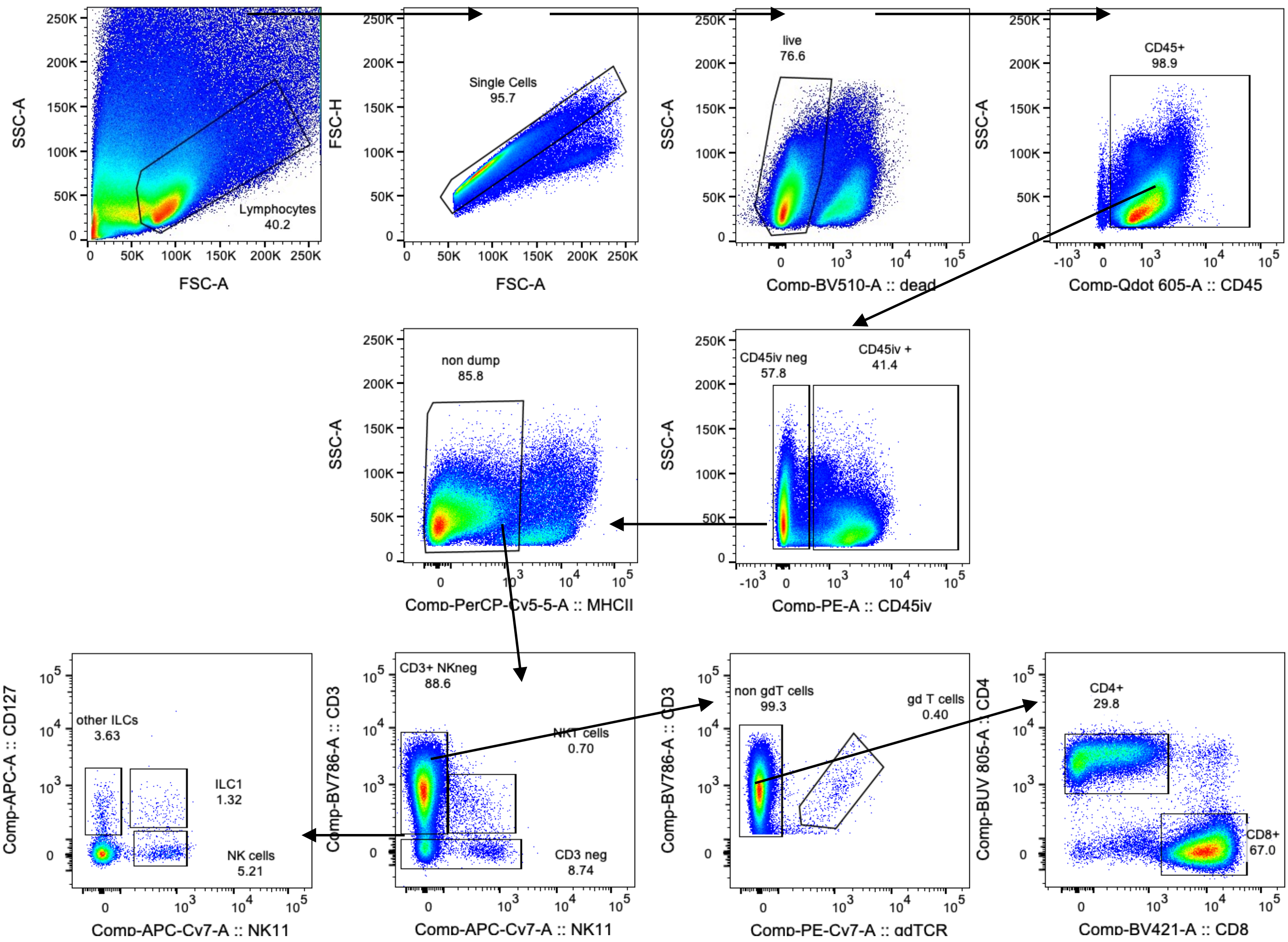

Supplementary Figure 1

Gating strategy diagram for flow cytometry analysis of immune cell populations in IAV-infected mouse lung

GREATxSMART mice were infected with IAV on day 0 and injected with fluorescently labelled anti-CD45 i.v. 3 minutes prior to removal of the tissues. Representative flow for NK cells (CD3-, NK1.1+, CD127-), NK T cells (CD3+, NK1.1+), ILC1s (CD3-, NK1.1+, CD127+) other ILCs (CD3-, NK1.1-, CD127+), CD4 (CD3+, $\gamma\delta$ TCR-, CD4+, CD8-) CD8 (CD3+, $\gamma\delta$ TCR-, CD4-, CD8+) and $\gamma\delta$ T cells (CD3+, $\gamma\delta$ TCR+) 10 days following infection in the lung. Numbers indicate percentage of cells within each gate.

Supplementary Figure 2

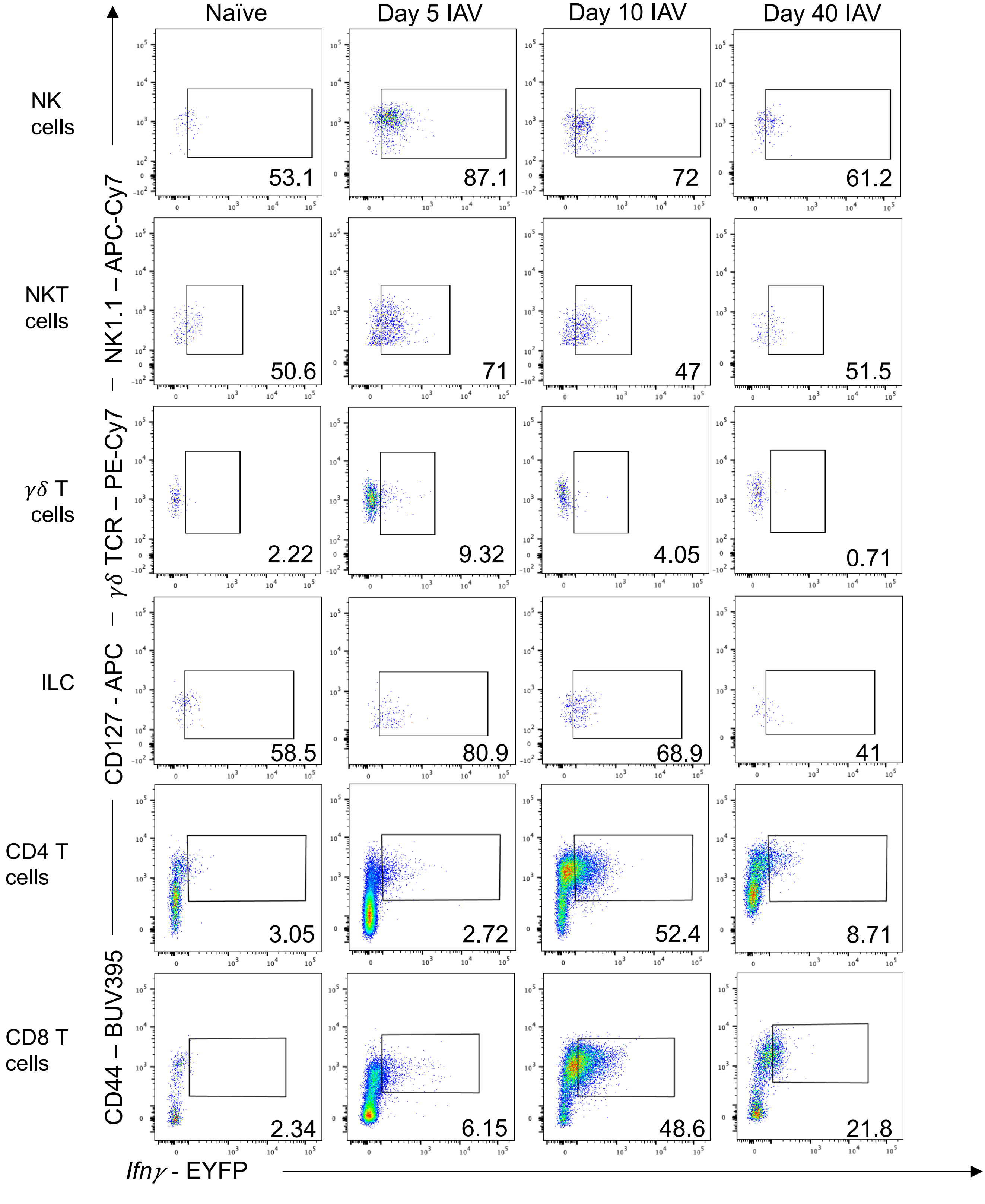

Supplementary Figure 2

Characterisation of Ifnγ *expression from immune cells during IAV infection in the lung*

GREATxSMART mice were infected with IAV on day 0 and injected with fluorescently labelled anti-CD45 i.v. 3 minutes prior to removal of the tissues. Single cell suspensions of lungs were examined after 5, 10 or 40 days to examine *Ifnγ* transcription of indicated cell populations determined by EYFP+ and CD45iv$^-$ populations compared to naïve. Numbers indicate percentage of *Ifnγ*$^+$ populations of each cell type.

Supplementary Figure 3

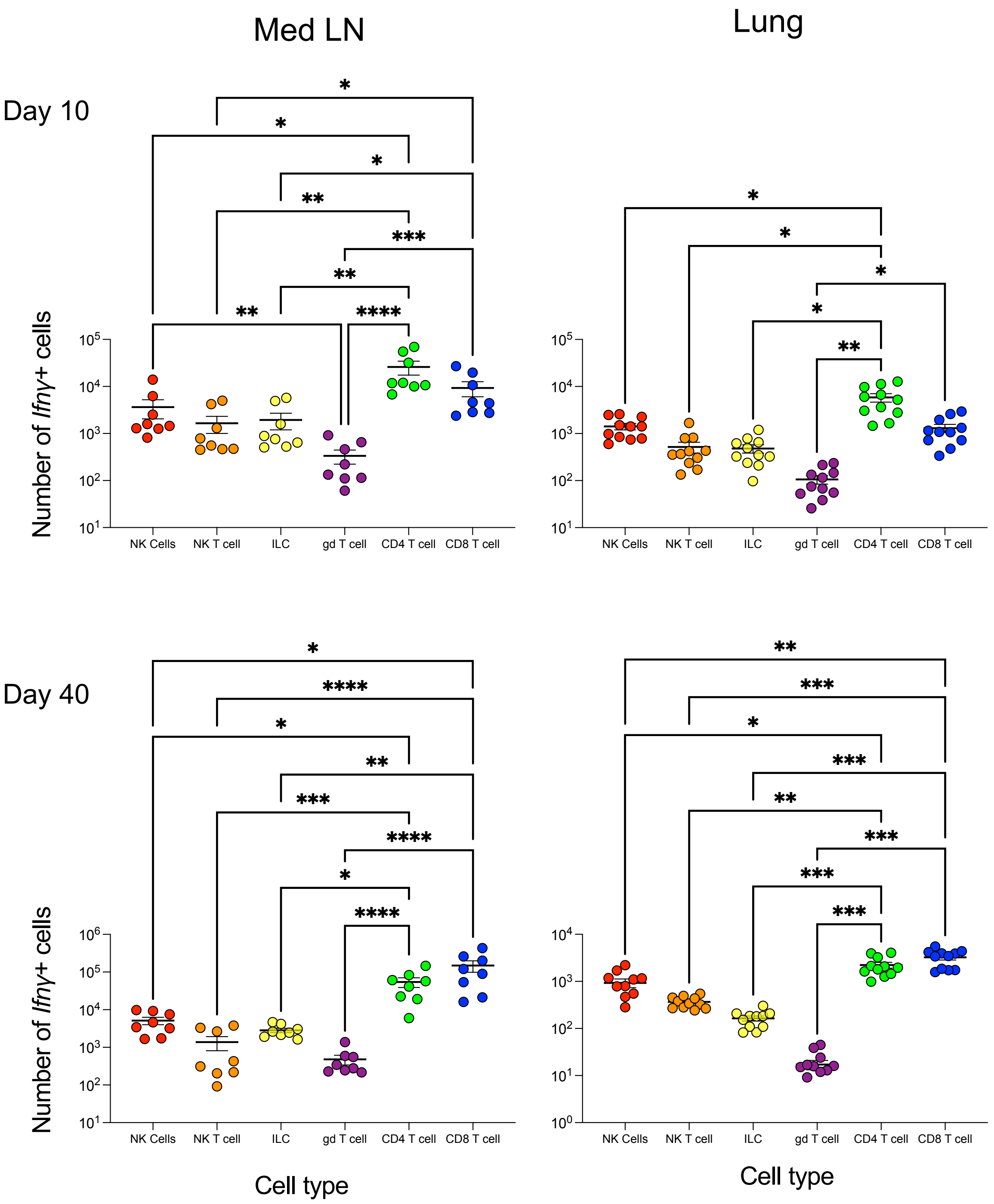

Supplementary Figure 3

CD4 and CD8 T cells are the predominant source of Ifnγ *10 and 40 days after IAV infection in the lung and lymph node*

GREATxSMART mice were infected with IAV on day 0 and injected with fluorescently labelled anti-CD45 i.v. 3 minutes prior to removal of the tissues. The total number of *Ifnγ*+ cells of the indicated cell populations in the Med LN and lung were examined in animals infected 10 (top) or 40 (bottom) days previously. Each point represents an individual mouse of 8-11 infected mice from two independent time course experiments; 8-11 mice are combined from across the time points and experiments, error bars are SEM. All statistics were calculated using a Shapiro-Wilk and Kruskal-Wallis test. , * = <0.05, ** = <0.01, *** = <0.001, **** = <0.0001. Some Med LN samples were removed from the analysis for technical reasons.

Supplementary Figure 4

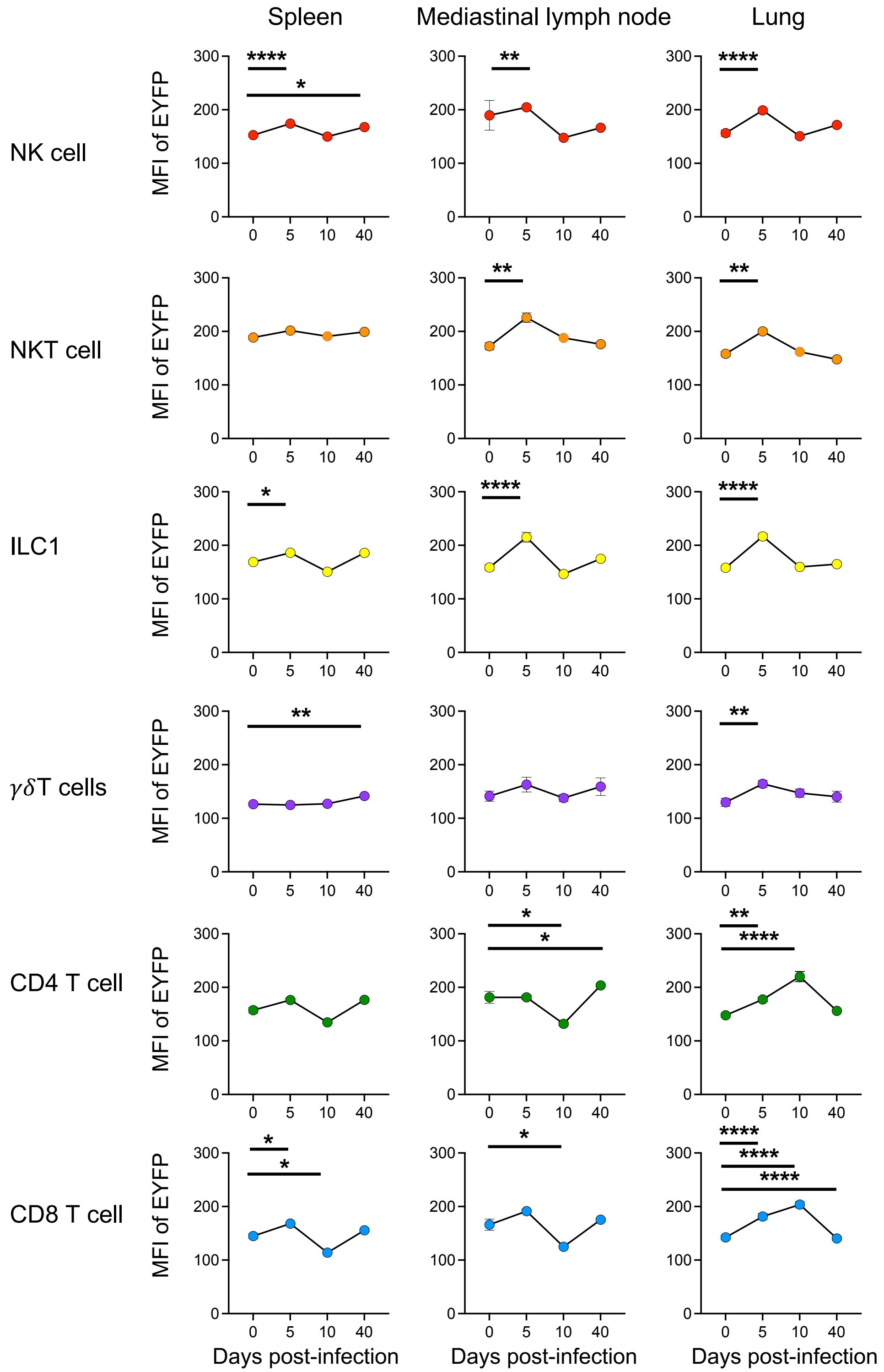

Supplementary Figure 4

The amount of immune cell-derived Ifnγ *fluctuates between the tissue and secondary lymphoid organs*

GREATxSMART mice were infected with IAV on day 0 and injected with fluorescently labelled anti-CD45 i.v. 3 minutes prior to removal of the spleen, Med LN and lung. The mean fluorescence intensity (MFI) of the indicated cell populations were examined in naïve animals or those infected 5, 10 or 40 days previously. Each point represents the mean of 8-11 infected mice from two independent time course experiments; 21 naïve mice are combined from across the time points and experiments, error bars are SEM. Significance tested via a Kruskal–Wallis test followed by a Dunn's multiple comparison test; * = <0.05, ** = <0.01, **** = <0.0001.

Supplementary Figure 5

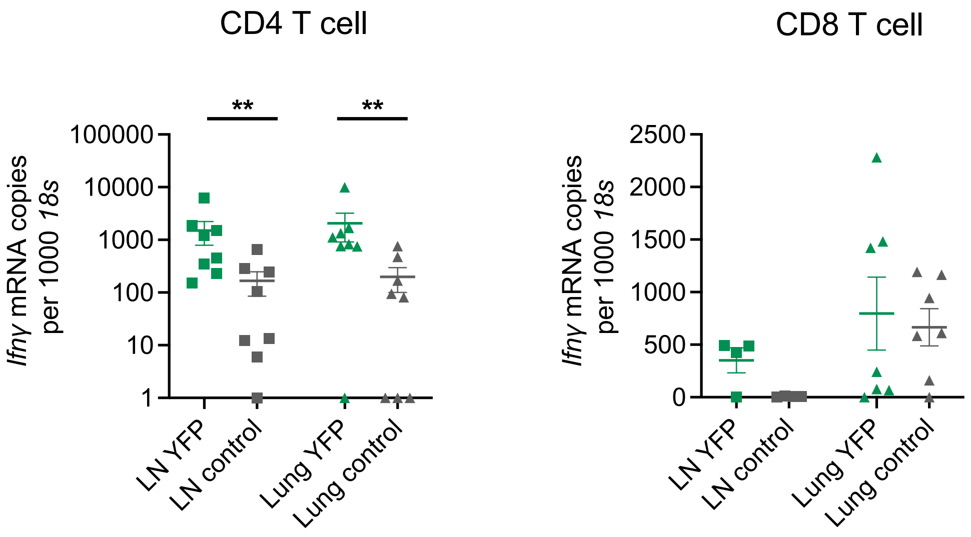

Supplementary Figure 5

EYFP expression reports Ifnγ *expression in CD4 T cells after IAV infection*

GREATxSMART mice were infected with 200PFU IAV i.n and EYFP+ and EYFP- T cells were FACS sorted from the Med LN and lung of mice 40 days after infection. Gene expression of *Ifnγ* by CD4 (left) and CD8 (right) T cells in the lymph node (LN) and lung of IAV-infected mice shown. All gene expression was standardised against the housekeeping gene (*18S*) and is presented as the change in absolute copy number of transcripts. Each point represents an individual mouse and data are combined from two independent experiments; error bars are SEM. Significance tested via a Shapiro-Wilk normality test and Wilcoxon test. ** = <0.01.

Table 1

Spleen

NK cell

	Day 40	Day 10	Day 5	Naïve
Naïve	ns	ns	**	
Day 5	****	ns		
Day 10	*			
Day 40				

NK T cell

	Day 40	Day 10	Day 5	Naïve
Naïve	ns	****	ns	
Day 5	ns	****		
Day 10	****			
Day 40				

ILC1

	Day 40	Day 10	Day 5	Naïve
Naïve	ns	**	ns	
Day 5	ns	*		
Day 10	***			
Day 40				

$\gamma\delta$ T cell

	Day 40	Day 10	Day 5	Naïve
Naïve	ns	****	**	
Day 5	ns	ns		
Day 10	*			
Day 40				

CD4 T cell

	Day 40	Day 10	Day 5	Naïve
Naïve	ns	**	*	
Day 5	**	ns		
Day 10	**			
Day 40				

CD8 T cell

	Day 40	Day 10	Day 5	Naïve
Naïve	ns	****	****	
Day 5	***	ns		
Day 10	ns			
Day 40				

Mediastinal LN

NK cell

	Day 40	Day 10	Day 5	Naïve
Naïve	*	**	****	
Day 5	ns	ns		
Day 10	ns			
Day 40				

NK T cell

	Day 40	Day 10	Day 5	Naïve
Naïve	*	***	****	
Day 5	ns	ns		
Day 10	ns			
Day 40				

ILC1

	Day 40	Day 10	Day 5	Naïve
Naïve	*	****	****	
Day 5	ns	ns		
Day 10	ns			
Day 40				

$\gamma\delta$ T cell

	Day 40	Day 10	Day 5	Naïve
Naïve	**	**	****	
Day 5				
Day 10				
Day 40				

CD4 T cell

	Day 40	Day 10	Day 5	Naïve
Naïve	**	***	****	
Day 5	ns	ns		
Day 10	ns			
Day 40				

CD8 T cell

	Day 40	Day 10	Day 5	Naïve
Naïve	**	***	****	
Day 5	ns	ns		
Day 10	ns			
Day 40				

Lung

NK cell

	Day 40	Day 10	Day 5	Naïve
Naïve	ns	*	**	
Day 5	ns	ns		
Day 10	ns			
Day 40				

NK T cell

	Day 40	Day 10	Day 5	Naïve
Naïve	ns	***	*	
Day 5	ns	ns		
Day 10	ns			
Day 40				

ILC1

	Day 40	Day 10	Day 5	Naïve
Naïve	ns	***	*	
Day 5	ns	ns		
Day 10	**			
Day 40				

$\gamma\delta$ T cell

	Day 40	Day 10	Day 5	Naïve
Naïve	ns	ns	ns	
Day 5	ns	ns		
Day 10	ns			
Day 40				

CD4 T cell

	Day 40	Day 10	Day 5	Naïve
Naïve	ns	****	ns	
Day 5	ns	ns		
Day 10	ns			
Day 40				

CD8 T cell

	Day 40	Day 10	Day 5	Naïve
Naïve	ns	****	*	
Day 5	ns	ns		
Day 10	ns			
Day 40				

Supplementary Table 1

Characterization of immune cells during IAV infection

Significance tables from spleen, draining lymph node and lungs from mice infected with IAV, comparing significantly different numbers of NK cells, NK T cells, ILC1s, $\gamma\delta$ T cells, CD4 and CD8 T cells. Significance tested via a Kruskal–Wallis test followed by a Dunn's multiple comparison test, ns = non-significant, *: $p<0.05$, **: $p<0.01$, ***: $p<0.001$, ****: $p<0.0001$.

Table 2

Spleen

NK cell

	Day 40	Day 10	Day 5	Naïve
Naïve	ns	ns	**	
Day 5	****	ns		
Day 10	*			
Day 40				

NK T cell

	Day 40	Day 10	Day 5	Naïve
Naïve	ns	**	ns	
Day 5	ns	ns		
Day 10	ns			
Day 40				

ILC1

	Day 40	Day 10	Day 5	Naïve
Naïve	ns	ns	ns	
Day 5	ns	ns		
Day 10	ns			
Day 40				

$\gamma\delta$ T cell

	Day 40	Day 10	Day 5	Naïve
Naïve	ns	*	****	
Day 5	**	ns		
Day 10	ns			
Day 40				

CD4 T cell

	Day 40	Day 10	Day 5	Naïve
Naïve	ns	ns	ns	
Day 5	ns	ns		
Day 10	ns			
Day 40				

CD8 T cell

	Day 40	Day 10	Day 5	Naïve
Naïve	**	*	ns	
Day 5	ns	ns		
Day 10	ns			
Day 40				

Mediastinal LN

NK cell

	Day 40	Day 10	Day 5	Naïve
Naïve	*	**	****	
Day 5	ns	ns		
Day 10	ns			
Day 40				

NK T cell

	Day 40	Day 10	Day 5	Naïve
Naïve	*	**	****	
Day 5	ns	ns		
Day 10	ns			
Day 40				

ILC1

	Day 40	Day 10	Day 5	Naïve
Naïve	*	***	****	
Day 5	ns	ns		
Day 10	ns			
Day 40				

$\gamma\delta$ T cell

	Day 40	Day 10	Day 5	Naïve
Naïve	ns	**	****	
Day 5	ns	ns		
Day 10	ns			
Day 40				

CD4 T cell

	Day 40	Day 10	Day 5	Naïve
Naïve	***	****	*	
Day 5	ns	ns		
Day 10	ns			
Day 40				

CD8 T cell

	Day 40	Day 10	Day 5	Naïve
Naïve	*	****	****	
Day 5	ns	ns		
Day 10	ns			
Day 40				

Lung

NK cell

	Day 40	Day 10	Day 5	Naïve
Naïve	ns	*	**	
Day 5	*	ns		
Day 10	*			
Day 40				

NK T cell

	Day 40	Day 10	Day 5	Naïve
Naïve	ns	ns	**	
Day 5	ns	ns		
Day 10	ns			
Day 40				

ILC1

	Day 40	Day 10	Day 5	Naïve
Naïve	ns	**	*	
Day 5	*	ns		
Day 10	**			
Day 40				

$\gamma\delta$ T cell

	Day 40	Day 10	Day 5	Naïve
Naïve	ns	***	***	
Day 5	**	ns		
Day 10	**			
Day 40				

CD4 T cell

	Day 40	Day 10	Day 5	Naïve
Naïve	**	****	ns	
Day 5	ns	ns		
Day 10	ns			
Day 40				

CD8 T cell

	Day 40	Day 10	Day 5	Naïve
Naïve	**	****	ns	
Day 5	ns	ns		
Day 10	ns			
Day 40				

Supplementary Table 2

Characterization of IFNγ-expressing cells during IAV infection

Significance tables from spleen, draining lymph node and lungs from mice infected with IAV, comparing significantly different numbers of IFNγ+ NK cells, NK T cells, ILC1s, $\gamma\delta$ T cells, CD4 and CD8 T cells. Significance tested via a Kruskal–Wallis test followed by a Dunn's multiple comparison test, ns = non-significant, * = <0.05, ** = <0.01, *** = <0.001, **** = <0.0001.

Table 3

Spleen

NK cell

	Day 40	Day 10	Day 5	Naïve
Naïve	*	ns	****	
Day 5	ns	****		
Day 10	*			
Day 40				

NK T cell

	Day 40	Day 10	Day 5	Naïve
Naïve	ns	ns	ns	
Day 5	ns	ns		
Day 10	ns			
Day 40				

ILC

	Day 40	Day 10	Day 5	Naïve
Naïve	ns	ns	*	
Day 5	ns	***		
Day 10	***			
Day 40				

$\gamma\delta$ T cell

	Day 40	Day 10	Day 5	Naïve
Naïve	**	ns	ns	
Day 5	**	ns		
Day 10	*			
Day 40				

CD4 T cell

	Day 40	Day 10	Day 5	Naïve
Naïve	ns	ns	ns	
Day 5	ns	**		
Day 10	**			
Day 40				

CD8 T cell

	Day 40	Day 10	Day 5	Naïve
Naïve	ns	*	*	
Day 5	ns	****		
Day 10	*			
Day 40				

Mediastinal LN

NK cell

	Day 40	Day 10	Day 5	Naïve
Naïve	ns	ns	**	
Day 5	ns	ns		
Day 10	****			
Day 40				

NK T cell

	Day 40	Day 10	Day 5	Naïve
Naïve	ns	ns	**	
Day 5	**	ns		
Day 10	ns			
Day 40				

ILC

	Day 40	Day 10	Day 5	Naïve
Naïve	ns	ns	****	
Day 5	****	****		
Day 10	**			
Day 40				

$\gamma\delta$ T cell

	Day 40	Day 10	Day 5	Naïve
Naïve	ns	ns	ns	
Day 5	ns	ns		
Day 10	ns			
Day 40				

CD4 T cell

	Day 40	Day 10	Day 5	Naïve
Naïve	*	*	ns	
Day 5	ns	ns		
Day 10	****			
Day 40				

CD8 T cell

	Day 40	Day 10	Day 5	Naïve
Naïve	ns	*	ns	
Day 5	ns	***		
Day 10	ns			
Day 40				

Lung

NK cell

	Day 40	Day 10	Day 5	Naïve
Naïve	ns	ns	****	
Day 5	ns	***		
Day 10	ns			
Day 40				

NK T cell

	Day 40	Day 10	Day 5	Naïve
Naïve	ns	ns	**	
Day 5	****	ns		
Day 10	ns			
Day 40				

ILC

	Day 40	Day 10	Day 5	Naïve
Naïve	ns	ns	****	
Day 5	**	**		
Day 10	ns			
Day 40				

$\gamma\delta$ T cell

	Day 40	Day 10	Day 5	Naïve
Naïve	ns	ns	**	
Day 5	ns	ns		
Day 10	ns			
Day 40				

CD4 T cell

	Day 40	Day 10	Day 5	Naïve
Naïve	ns	****	**	
Day 5	ns	ns		
Day 10	**			
Day 40				

CD8 T cell

	Day 40	Day 10	Day 5	Naïve
Naïve	***	****	****	
Day 5	****	ns		
Day 10	***			
Day 40				

Supplementary Table 3

Characterization of the mean fluorescence intensity of IFNγ produced by cells during IAV infection

Significance tables from spleen, draining lymph node and lungs from mice infected with IAV, comparing significantly different mean fluorescence intensity (MFI) of IFNγ+ NK cells, NK T cells, ILCs, $\gamma\delta$ T cells, CD4 and CD8 T cells. Significance tested via a Kruskal–Wallis test followed by a Dunn's multiple comparison test, ns = non-significant, * = <0.05, ** = <0.01, *** = <0.001, **** = <0.0001.
